# Macrophage migration inhibitory factor promoter polymorphisms (−794 CATT5–8): Relationship with soluble MIF levels in coronary atherosclerotic disease subjects

**DOI:** 10.1186/s12872-017-0570-x

**Published:** 2017-06-02

**Authors:** Lu Qian, Xiao-Yan Wang, Saroj Thapa, Lu-yuan Tao, Shao-Ze Wu, Gao-Jiang Luo, Lu-Ping Wang, Jiao-Ni Wang, Jie Wang, Ji Li, Ji-Fei Tang, Kang-Ting Ji

**Affiliations:** 1 Department of Cardiology, the Second Affiliated Hospital, Wenzhou Medical University, Wenzhou, Zhejiang, 325000 China; 2 Department of Cardiology, Yiwu Central Hospital, Yiwu, 322000 China

**Keywords:** Macrophage Migration Inhibitory Factor, Gene polymorphisms, Coronary Atherosclerotic Disease, Gensini’s degree integral

## Abstract

**Background:**

We analyzed the relationship of −794 CATT5–8 MIF polymorphisms with soluble MIF in Coronary Atherosclerotic Disease (CAD) patients.

**Methods:**

A total of 256 patients selected, on which 186 normal-coronary and 70 Coronary artery disease subjects, were recruited in the study (Retrospectively registered). Genotyping of −794 CATT5–8 polymorphisms were performed by PCR and DNA sequencing. Serum MIF levels were measured using an ELISA kit. Patients were classified by coronary angiogram, and CAD based on Gensini’s integral degree (angiographic scoring system).

**Results:**

The allele frequency and genotype frequency of −794 CATT5–8 did not show any differences in normal-coronary subjects and CAD subjects. In CAD patients, serum MIF levels was lower in CATT (5) subjects than in CATT (7) subjects, while the genotype of −794 CATT5–8 did not show differences in serum MIF levels. In addition, we found a decrease in serum MIF levels in carriers of the (5/5) genotypes the −794 CATT5–8 MIF polymorphisms, although it was not significant. There was no relationship of CAD class and the allele frequency of −794 CATT5–8.

**Conclusions:**

This study found no association between CAD class and −794 CATT5–8 MIF polymorphisms with soluble MIF levels in CAD Subjects.

**Trial registration:**

NCT01750502 (November 2012, Retrospectively registered).

## Background

Coronary Atherosclerotic Disease (CAD) is characterized by atherosclerotic plaques in the vascular wall that results in vascular stenosis or plaque disruption with acute thrombotic occlusion. It occurs due to gradual cholesterol and fibrous tissue plaque in the wall of coronary artery over long period [1]. Various risk factors have been identified for CAD, such as smoking, hypercholesterolemia, hypertension, and diabetes [2].

Evidence suggests that CAD is an inflammatory process with chronic inflammation of vessel wall infiltrated by circulating immune cells, such as monocytes and macrophages [3]. Macrophage migration inhibitory factor (MIF) is a homotrimer protein with a molecular weight of 37.5 kDa, which can promote the inflammation [4]. The first experimental studies that utilized pure recombinant MIF and neutralizing antibodies established that MIF played a critical role in the inflammatory cascade leading to endotoxic shock and death [5] . Soon thereafter, it was found that the macrophage, which had been considered historically to be the “target” of MIF action, was in fact a significant source of MIF production. In the case of the macrophage, MIF promotes TNFα production, which leads to further MIF release and a re-entrant activation pathway that is required for the optimal expression of TNFα and other pro-inflammatory mediators [6]. MIF is expressed in several cell types, including monocytes, macrophages, vascular smooth muscle cells (SMCs), and cardiomyocytes [6–8].

As described by Jie Wu et al. [9], the MIF gene maps to chromosome 21q22.33 in human (2119 bp). There are four polymorphisms that have been mainly reported in the human MIF gene, including three Single Nucleotide Polymorphisms (SNPs) at positions −173 (rs755622), +254 (rs2096525), and +656 (rs2070766) and a 794CATT5–8 microsatellite polymorphism. Loci rs2096525 and rs2070766 are located in introns, whereas rs755622 and −794CATT5–8 are located in the promoter region of MIF [9] . Earlier studies have found that circulating MIF levels are elevated in ulcerative colitis (UC) [10], psoriasis [9] and tuberculosis (TB) [11]. Since these diseases are accompanied by persistent inflammation of varying degrees, it is possible that MIF may play a role in the development of these diseases.

Previous studies have reported that the plasma MIF level of CAD group was higher than non-CAD patients and the plasma MIF level was related to the stability of the plaque [12]. Also, we demonstrated a close association between the polymorphism of MIF on the −173 position and CAD [12]. The aim of this study was to investigate the relationship between −794 CATT5–8 MIF polymorphisms and soluble MIF levels in CAD patients.

## Methods

### Subjects

A total of 256 subjects were enrolled, including 186 without CAD and 70 with CAD subjects, in the period from 06/2012 to 12/2012 in our inpatient department. All patients underwent coronary angiography (CAG) interpreted by one independent radiologist. Stenosis of the left main artery [13], Left Anterior Descending (LAD) branch, Right Coronary Artery (RCA), and other major branches were evaluated. CAD patients had the evidence of atherosclerosis (i.e., ≥ 50% luminal stenosis) in at least one coronary artery or major branch segment in their epicardial coronary tree. Patients in the control group had no luminal stenosis at CAG. Patients were excluded if they had acute inflammatory diseases, tumors, autoimmune disease and severe hepatic and renal dysfunction. All participants were of Han ethnicity living in Wenzhou, a southeastern coastal city of China. This protocol was approved by the Research Ethics Committee of Wenzhou Medical University (registration number L-2013-03). All authors have identified individual participants after data collection.

### Gensini score

The severity of CAD was determined by Gensini scoring system which has been previously described [14]. Briefly, if any branches of main coronary artery Left Main Artery (LM), LAD, Left Circumflex Coronary Artery (LCX) and RCA has stenosis reaching 1–24% of the internal lumen diameter, 1 point is given. Similarly, 2 is given for 25–49% stenosis, 4 for 50–74%, 8 for 75–90%, 16 for 91–99% and 32 for 100% or total occlusion. Depending on the lesion location, the single lesion score and the coefficient, the final Gensini total score was calculated.

### Human genomic DNA extraction

A blood sample of 5 mL was collected into a tube containing ethylene diamine tetra acetic acid (EDTA) from the radial artery. After centrifugation, plasma was collected and stored at −80 °C until use. Genomic DNA was extracted from cells by a DNA extraction kit (Tiangen Company, Beijing, China). The isolated DNA was also stored at −80 °C.

### MIF − 794 CATT5–8 genotyping

Polymorphism was genotyped by sequencing of polymerase chain reaction (PCR) product as reported previously [4, 15]. The forward primer was 5-TTGCACCTATCAGAGACC-3 and the reverse primer was 5-TCCACTAATGGTAAACTCG-3. These primers were designed to amplify a 207 bp segment of the MIF promoter region. PCR was carried out in a volume of 25 μl. The reaction conditions of PCR were as follows: initial denaturation at 95 °C for 5 min, followed by 35 cycles at 95 °C for 30 s, 60 °C for 30 s, and 72 °C for 1 min, with final extension at 72 °C for 10 min. PCR products were revealed by agarose gel electrophoresis.

Genomic DNA was extracted from blood collected into tubes containing EDTA. The DNA of individuals previously sequenced was used as a template to generate control DNA fragments, using BigDye Terminator v1.1, in order to correlate the fragment size observed on the ABI 310 analyzer with the number of CATT repeats in the test samples [16]. (Sequenced by Shanghai Hybio BioTechnology Co., Ltd.).

### Analysis of serum MIF levels

The plasma concentrations of MIF were measured using an enzyme linked immunosorbent assay (ELISA) kit according to the manufacturer’s instructions (R&D, USA).

### Statistic analyses

MIF genotype and allele frequencies were analyzed using SPSS17.0 statistical software. The allele and genotype distributions were estimated by gene counting, and distribution of the polymorphic variants was tested against Hardy–Weinberg (H–W) equilibrium by χ2 analysis. Plasma MIF concentrations were expressed as means ± SD. For comparisons between two groups, we determined the significance of differences between means by t-tests. Comparisons between multiple groups were performed by ANOVA. *P* ≤ 0.05 was considered statistically significant.

## Results

### Frequencies of MIF − 794 alleles and genotypes of CAD patients and controls

There were no significant differences in age, cigarette smoking, drinking hypertension, and diabetes except gender, (*P* > 0.05) (Table 1) between the CAD patients and the control. Both CAD patients and controls were in Hardy-Weinberg equilibrium with MIF −794CATT5–8 genotypes’ distribution (*P* > 0.05). There were seven kinds of genotypes and four kinds of alleles in these two groups (Table 2). The comparative analysis of genotype and allele frequencies of −794 CATT5–8 polymorphisms between groups did not show significant differences (*P* > 0.05).

**Table 1:** Clinical and biochemical characteristics by study group

	Control group (*n* = 186)	CHD group (*n* = 70)	F/×^2^	*P* value
Mean age(year)^a^	60.78 ± 9.26	66.71 + 10.25	1.24	0.081
Gander^b^ (male/female)	84/102	44/26	6.37	0.012
cigarette smoking(%)^b^	31.6%	33.3%	0.236	0.627
Drinking(%)^b^	18.3%	24.3%	0.677	0.41
Hypertension(%)^b,c^	66.7%	68.6%	0.054	0.817
Hyperlipidemia(%)^b,d^	16.7%	37.1%	6.76	0.009
Diabetes(%)^b,e^	3.3%	7.1%	0.92	0.337

^a^Data presented as mean ± SD. Student’s t-test

^b^Chi-square

^c^blood pressure ≥ 140/90 mmHg

^d^LDL-C ≥ 120mg/dl

^e^FPG ≥ 7.0 mmol/l and/or OGTT 2 h FPG ≥ 11.1 mmol/l

**Table 2:** Genotype and allele frequencies of −794 CATT5–8 MIF polymorphisms

Polymorphisms	Genotypes/alleles	CHD group *n* = 70	Control group *n* = 186	x^2^	*P* value^a^
-794CATT	5/5	11(15.7)	31(17.6)		
	6/6	16(22.9)	30(17.0)		
	7/7	3(4.3)	7(4.0)		
	5/6	22(31.4)	56(31.8)		
	5/7	10(14.3)	24(13.6)		
	6/7	8(11.4)	26(14.8)		
	6/8	0(0)	1(0.54)	0.464	0.834
	5	54(38.6)	132(35.5)		
	6	62(44.3)	190(51.1)		
	7	24(17.1)	49(13.2)		
	8	0(0)	1(0.27)	0.003	0.959

^a^Chi-square test χ^2^

### The plasma concentration of MIF

The plasma MIF concentration of CAD group was 65.75 ± 6.32 μg/L, significantly higher than that of non-CAD group (51.13 ± 7.33μg/L, *P* < 0.05), as we known before [12]. MIF serum levels were similar among CATT (5), CATT (6), and CATT (7) allele carriers. (Table 3).

**Table 3:** The plasma MIF concentration

	CHD group (n = 70)	Control group (n = 186)	P* value	
mean concentration of MIF + SD	65.75 ± 6.32	51.13 ± 7.33		
median of MIF	65.66	50.01	0.00	
	alleles 5 (2n = 186)	alleles 6 (2n = 252)	alleles 7 (2n = 73)	P^#^ value
mean concentration of MIF + SD	58.68 + 7.80	60.22 + 9.77	62.09 + 5.32	
median of MIF	58.275 + 1.42	59.75 + 0.98	61.79 + 0.51	0.373

*Student’s t-test

^#^Chi-square test x^2^

**Fig. 1:**
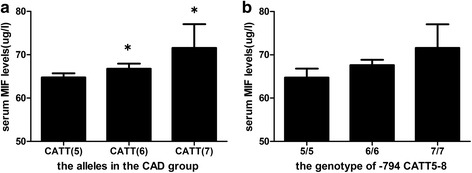
the plasma MIF concentration of carriers of MIF −794CATT5–8 alleles and genotypes in CAD patients. **a** The plasma MIF concentration of the carriers of CATT(5) allele was significantly lower than that of the CATT(7) allele carriers (*P* < 0.05). **b** The plasma MIF concentration was lower in the CATT(5/5) group than CATT(6/6) and CATT(7/7) groups but the difference was not statically significant (*P* > 0.05)

In CAD patients the plasma MIF concentration of the carriers of CATT(5) allele was significantly lower than that of the CATT(7) allele carriers (*P* < 0.05) (Fig. 1a). When MIF serum levels were compared among CAD patients with different genotypes, we did not observe significant difference. In the CAD patients, the plasma MIF concentration was lower in the CATT(5/5) group than CATT(6/6) and CATT(7/7) groups but the difference was not statically significant (*P* > 0.05) (Fig. 1b). While in normal-coronary subjects, we did not observe a correlation between MIF serum levels with allele and genotypes (*P* > 0.05) (data not shown).

### Frequencies of MIF− CATT_5–8_ alleles among CAD patients

Patients were classified into three subgroups according to the Gensini system. There was no difference in MIF− CATT5–8 allele frequency in CAD patients with different scores (Table 4).

**Table 4:** the relationship between CHD class and the allele frequency of MIF-794 CATT_5−8_

CHD class (Gensini’s intergral degree)	the allele frequency of MIF-794 CATT_5–8_	
	5	6
≦20	16	10
20–40	14	10
≧40	13	4
x^2^	1.55	
P^a^ value	0.46	

^a^Chi-square test

## Discussion

Despite the improvements in medical treatments and subsequent survival rates, CAD is still the leading cause of death worldwide [1]. In addition, it is well documented that there is strong relationship between many genetic variants and environmental factor in CAD. Therefore, the knowledge of genetic mechanisms of CAD is helpful to develop new disease prevention and treatment strategies.

Polymorphism of MIF on the −173 position has been reported in several inflammatory diseases, including ulcerative colitis (UC) [10], psoriasis [9] and tuberculosis (TB) [11]. Several studies have reported the association between MIF794CATT gene polymorphism, MIF protein level and CAD. For example in Western Mexico, MIF794CATT (6/7) genotype was found to correlate with the onset of acute coronary syndrome [17]. Lan et al. reported that discovered polymorphism of the MIF gene was associated with the severity of carotid atherosclerotic plaque [18]. In the present study, MIF794CATT gene polymorphism and CAD incidence were not significantly correlated, but in the CAD patients, CATT (5) allele carriers had lower serum MIF concentration than the other two groups. Indeed, in vitro studies found that, CATT (5) allele has the lowest level of basic and stimulated MIF promoter activity when compared to the CATT (6) and CATT (7) alleles [16, 19]. However, it is still unknown which transcription factors regulate the expression of MIF by binding to CATT gene promoter region [20].

We found that serum MIF concentrations were significantly higher in CAD patients than the non-CAD patients, which is inconsistence with the previous finding that increased incidence MIF protein concentration is related to increased incidence of CAD [12]. However, MIF794CATT5–8 allele were not associated with the severity of CAD.

Our study is based at single center and the cases are limited because of time and geographical restrictions. Further studies with bigger sample size and patients from more cities are needed to confirm this primary conclusion.

## Conclusion

To sum up, there is no significant correlation between the polymorphism of 794CATT gene and the severity of CAD.

## Abbreviations

CAD: Coronary Atherosclerotic Disease
CAG: Coronary Angiography
EDTA: Ethylene Diamine Tetra Acetic Acid
ELISA: Enzyme Linked Immunosorbent Assay
LAD: Left Anterior Descending
LCX: Left Circumflex Coronary Artery
LM: Left Main Artery
MIF: Migration Inhibitory Factor
PCR: Polymerase Chain Reaction
RCA: Right Coronary Artery
SMCs: Vascular smooth muscle cells
SNPs: Single Nucleotide Polymorphism
TB: Tuberculosis
UC: Ulcerative Colitis
